# A Prospective Study of LINE-1DNA Methylation and Development of Adiposity in School-Age Children

**DOI:** 10.1371/journal.pone.0062587

**Published:** 2013-04-30

**Authors:** Wei Perng, Mercedes Mora-Plazas, Constanza Marín, Laura S. Rozek, Ana Baylin, Eduardo Villamor

**Affiliations:** 1 Department of Epidemiology, University of Michigan School of Public Health, Ann Arbor, Michigan, United States of America; 2 Fundación para Investigación en Nutrición y Salud, FINUSAD; Bogotá, Colombia; 3 Department of Environmental Health Sciences, University of Michigan School of Public Health, Michigan, United States of America; Dartmouth Medical School, United States of America

## Abstract

**Background:**

Repetitive element DNA methylation is related to prominent obesity-related chronic diseases including cancer and cardiovascular disease; yet, little is known of its relation with weight status. We examined associations of LINE-1 DNA methylation with changes in adiposity and linear growth in a longitudinal study of school-age children from Bogotá, Colombia.

**Methods:**

We quantified methylation of LINE-1 elements from peripheral leukocytes of 553 children aged 5–12 years at baseline using pyrosequencing technology. Anthropometric characteristics were measured periodically for a median of 30 months. We estimated mean change in three age-and sex-standardized indicators of adiposity: body mass index (BMI)-for-age Z-score, waist circumference Z-score, and subscapular-to-triceps skinfold thickness ratio Z-score according to quartiles of LINE-1 methylation using mixed effects regression models. We also examined associations with height-for-age Z-score.

**Results:**

There were non-linear, inverse relations of LINE-1 methylation with BMI-for-age Z-score and the skinfold thickness ratio Z-score. After adjustment for baseline age and socioeconomic status, boys in the lowest quartile of LINE-1 methylation experienced annual gains in BMI-for-age Z-score and skinfold thickness ratio Z-score that were 0.06 Z/year (*P* = 0.04) and 0.07 Z/year (*P* = 0.03), respectively, higher than those in the upper three quartiles. The relation of LINE-1 methylation and annual change in waist circumference followed a decreasing monotonic trend across the four quartiles (*P* trend = 0.02). DNA methylation was not related to any of the adiposity indicators in girls. There were no associations between LINE-1 methylation and linear growth in either sex.

**Conclusions:**

Lower LINE-1 DNA methylation is related to development of adiposity in boys.

## Introduction

Childhood obesity has reached epidemic proportions worldwide. Of special concern are countries undergoing the nutrition transition, including those in Latin America, that have experienced a marked increase in pediatric obesity rates in the last two decades [1]. Although researchers have identified dietary and lifestyle factors accountable for the rapid increase in childhood obesity, the biological mechanisms remain unclear. Epigenetic animal models indicate that maternal intake of methyl-donor micronutrients is protective against offspring obesity through increased DNA methylation in genomic regions that regulate satiety and metabolism [2], [3], [4]. Recent epidemiologic evidence suggests that aberrant changes in methylation of repetitive elements, such as long interspersed nucleotide element (LINE)-1, in peripheral white blood cells (WBC) are associated with prominent obesity-related diseases including cancer [5], diabetes [6], and cardiovascular disease [7], [8], [9]. Yet, there is little research on DNA methylation and weight status.

Current evidence on WBC DNA methylation and body size consists mostly of mixed findings from cross-sectional studies in adult populations [8], [10], [11], [12], [13], [14]. Some studies of maternal-infant dyads have been conducted to investigate relations of perinatal characteristics, including birth weight, with cord blood LINE-1 methylation [15], [16], [17]. One study reported that high or low birth weight, as well as premature birth were associated with significantly lower DNA methylation [16]. However, inference from perinatal studies is limited because it is not known whether functional consequences of these associations contribute to obesity in later life. A recent study that used data from two independent birth cohorts identified nine differentially methylated genes in cord blood that were associated with body composition at 9 years of age [18]. While such findings shed light on specific pathways involved in weight gain, associations with methylation of repetitive elements are also important to understand, as they may provide a global picture of genomic stability [19] which has health implications beyond the regulatory function of a single gene. Furthermore, because repetitive element methylation is sensitive to environmental cues [20], elucidating its relation with health outcomes could uncover viable avenues for intervention. Considering that early life weight status influences cardiometabolic morbidity in adulthood [21], it is critical to identify modifiable molecular mechanisms that underlie childhood obesity.

In this study, we examined the prospective relation of LINE-1 DNA methylation at recruitment into a cohort with changes in adiposity and linear growth indicators in a representative group of low- and middle-income children from Bogotá, Colombia, a country at the early stages of the nutrition transition.

## Methods

We conducted this study in the context of the Bogotá School Children Cohort (BSCC), a longitudinal investigation of nutrition and health among children from public schools in Bogotá, Colombia, ongoing since 2006. Details of the study design have been previously reported [22]. Briefly, we recruited a representative sample of 3,202 school children aged 5–12 years in February 2006 from public schools in Bogotá, using a random sampling strategy. The sample represents families from low- and middle-income socioeconomic backgrounds in the city, as the public school system enrolls the majority of children from these groups [23].

At the time of enrollment, we sent comprehensive self-administered questionnaires to parents (82% response). The questionnaires inquired about sociodemographic characteristics (including age, marital status, education, and socioeconomic level) as well as anthropometric measures of the mother (self-reported height and weight) and information about physical activity and sedentary habits of the child. In the proceeding weeks, trained research assistants visited the schools to obtain anthropometric measurements and a fasting blood sample from the children. We measured height without shoes to the nearest 1 mm using a wall-mounted portable Seca 202 stadiometer, and weight in light clothing to the nearest 0.1 kg on Tanita HS301 solar-powered electronic scales. We measured the subscapular and tricipital skinfold thicknesses to the nearest 0.5 mm using Slim Guide Skinfold Calipers (Creative Health Products, Inc. Plymouth, MI) according to standard protocols [24]. Follow-up anthropometric measurements were obtained in June and November 2006 and once yearly thereafter by visiting the schools or homes of the children when they were absent from school on the day of assessment. At these follow-up visits, we also measured waist circumference using a non-extensible measuring tape at the level of the umbilicus [24].

The parents or primary caregivers of all children gave written informed consent prior to enrollment into the study. The Ethics Committee of the National University of Colombia Medical School approved the study protocol, and the Institutional Review Board at the University of Michigan approved the use of data and samples from the study.

### Laboratory Methods

At the baseline assessment, phlebotomists obtained a blood sample from the children’s antecubital vein after an overnight fast. We collected samples in EDTA tubes, carried out a complete blood count, and separated plasma into an aliquot for micronutrient and inflammation biomarker determinations; details are described elsewhere [25]. DNA was isolated from the buffy coat using the QIAmp DNA Blood Mini Kit (Qiagen, Hilden, Germany) and cryopreserved until transportation to the University of Michigan for analyses.

### LINE-1 DNA Methylation Determinations

We carried out pyrosequencing-based DNA methylation analysis according to methods described by Tost and Gut [26]. Approximately 500 ng of DNA was bisulfite-converted using the EpiTect Bisulfite Kit (Qiagen). Bisulfite conversion of DNA deaminates unmethylated cytosine to uracil, which is read as a thymidine during polymerase chain reaction (PCR). Methylated cytosines (5-methylcytosine) are protected from bisulfite conversion and thus remain unchanged, resulting in genome-wide methylation-dependent differences in DNA sequence. We assessed LINE-1 DNA methylation through simultaneous PCR of the DNA LINE-1 elements, using primers designed towards consensus LINE-1 sequences that allow for the amplification of a representative pool of repetitive elements. PyroQ-CpG software (Qiagen) estimated the degree of methylation as the percentage of 5-methylcytosine (%5 mC) computed over the sum of methylated and unmethylated cytosines of four LINE-1 CpG sites. We ran all assays, starting with the bisulfite conversion, in duplicate. We excluded %5 mC site measurements that were more than 5 standard deviations above or below the raw mean LINE-1 methylation (<69 or >91%5 mC) from the analyses.

### Statistical Analysis

Of the 2816 (88%) cohort participants for whom specimens were available, we selected a random sample of 600 children for LINE-1 methylation determinations. Five hundred sixty-eight children had adequate DNA concentrations. Of them, 553 children who had valid anthropometric measurements at baseline and at least one additional follow-up measurement constituted the final study population. These children did not differ substantially from the rest of the BSCC in terms of nutritional status or sociodemographic characteristics at the baseline assessment.

Because the distribution of %5 mC at each LINE-1 site differed from individual to individual, we used a mixed effects linear regression model to create the final person-specific LINE-1 methylation variable. First, we took the average %5 mC for each site across the duplicate runs since within-site correlations were high [25]. We then fit a mixed effects model including a random intercept for %5 mC methylation at each site that was allowed to vary from person to person. Accordingly, the empirical best linear unbiased predictors (EBLUPs) from the random effects represent the between-person variation in DNA methylation. We added the EBLUPs to the population raw average %5 mC across the four LINE-1 sites. This method enabled us to incorporate the between-person variability of the underlying means for each LINE-1 site.

Next, we examined the relations of LINE-1 methylation with change in three indicators of adiposity: BMI-for-age Z-score (BMIZ) as an indicator of overall adiposity [27]; waist circumference Z-score as an indicator of central adiposity [28]; and subscapular-to-triceps skinfold thickness ratio Z-score as an indicator of truncal subcutaneous adiposity [29] over the 2.5 years of follow-up, separately for boys and girls. We also examined associations with height-for-age Z-score. Children’s BMI-for-age and height-for-age Z-scores were calculated with use of the sex-specific growth references for children 5–19 years from the World Health Organization [30]. We age-standardized waist circumference and the subscapular-to-triceps skinfold thickness ratio with the LMS method [31] using data from children 5–16 years of age in the third National Health and Nutrition Examination Survey (NHANES III) [32]. The LMS method summarizes the distribution of each anthropometric measurement by its median (M) and coefficient of variation (S), plus a measure of skewness based on the Box-Cox power (L) to normalize data. This approach is ideal for age-related standardization because it accounts for differential degrees of asymmetry in the distribution of an anthropometric measure within each stratum of age, rather than assuming the same skewness across age groups. We estimated mean changes in each of the adiposity indicators and height-for-age Z-score during follow-up and compared them across quartiles LINE-1 methylation with the use of mixed effects linear regression models for repeated measures. In each model, the anthropometric measurement was the outcome and the predictors included indicator variables for quartiles of LINE-1 methylation, age in decimal years, and interaction terms between the LINE-1 methylation indicator and age. These mixed models included random effects for the intercept and slope; an unstructured variance-covariance matrix was specified to account for within-child correlations of anthropometric measurements. For the waist circumference model, we did not include random effects for the slope because we only obtained measurements in the second and third years of follow-up; the change in waist circumference represented the change between the two measurements taken during follow-up. These methods do not require an even number of observations or that measurements be collected at exactly the same time in all subjects, thus we included all measurements available for every child in the analyses. Because non-linear associations seemed apparent in preliminary analyses, we also examined associations with a dichotomous indicator that represented the 1^st^ vs. 2–4^th^ quartiles of LINE-1 methylation.

In the multivariable models, we considered adjustment by nutritional and sociodemographic covariates that are associated with LINE-1 methylation in this population [25], as well as known predictors of child growth. We used empirical estimates of variance in all models to overcome deviations from the multivariate normality assumption.

We carried out all analyses with the use of the Statistical Analyses System software (version 9.3; SAS Institute Inc).

## Results

Mean ± SD age of children was 8.8±1.7 years; 45.9% were boys. Each child contributed a median of 4 measurements for BMI, height, and skinfold thicknesses, and a median of 2 measurements for waist circumference over a median of 30 months of follow-up (IQR = 29, 31). Prevalence of overweight/obesity at baseline (BMI-for-age Z-score >1) was 18.8%. Boys had 0.52%5 mC higher LINE-1 methylation than girls (*P*<0.0001). There was an inverse relation between LINE-1 methylation and socioeconomic status (**Table 1**). Additional correlates of LINE-1 methylation in this population have been reported before [25].

**Table 1:** Characteristics of 553 school-age children from Bogotá, Colombia by quartiles of LINE-1 methylation^1^.

	Q1	Q2	Q3	Q4
	N = 139	N = 138	N = 138	N = 138
Male, % (N)	36.7 (51)	38.4 (53)	51.5 (71)	57.3 (79)
Age, years	8.9±1.7	8.7±1.9	9.0±1.6	8.8±1.6
LINE-1 DNA methylation, %5 mC	78.36±0.94	79.80±0.24	80.67±0.27	82.17±0.97
Child was born in Bogota, % (N)^2^	88.3 (113)	92.7 (115)	88.1 (111)	93.0 (119)
Birthweight, g	3144±863	3263±887	3279±935	3126±690
Height-for-age Z-score^3^	−0.75±0.96	−0.80±1.06	−0.74±0.96	−0.83±0.92
BMI-for-age Z-score^3^	0.09±0.92	0.12±1.01	0.09±0.95	0.08±1.08
C-reactive protein, mg/L	1.25±1.44	1.31±1.76	2.09±5.49	1.32±2.64
Plasma vitamin A, µmol/L	1.08±0.33	1.02±0.34	1.02±0.32	1.03±0.35
Maternal Education, y	8.6±3.1	8.5±3.2	8.6±3.2	8.9±3.4
Maternal Height, cm	158.2±6.3	158.6±6.2	157.2±5.6	157.4±6.0
Maternal BMI, kg/m^2^	24.1±4.0	23.7±3.8	24.9±3.8	24.2±3.5
Lowest Socioeconomic Status, % (N)^4^	3.6 (5)	8.7 (12)	8.7 (12)	10.1 (14)

1 Values are mean ± SD unless otherwise noted.

2 Total is <553 due to missing values.

3 According to the World Health Organization 2007 growth reference for children 5–19 years [30].

4 Stratum 1 of a maximum of 4, according to the local government classification for tax and planning purposes.

Among boys, LINE-1 methylation was not related to baseline BMI-for-age Z-score or skinfold thickness ratio Z-score (*P* trend = 0.73 and *P* trend = 0.60, respectively). However, DNA methylation was inversely related to change in these indicators during follow-up in a non-linear manner (**Table 2**). Compared to boys in the upper three quartiles, those in the lowest quartile of LINE-1 methylation experienced a 0.06 Z/year (95% CI = −0.11, 0.00; *P = *0.04) higher annual gain in BMI-for-age Z-score, or approximately 0.1 kg/m^2^/year, after adjustment for baseline age and socioeconomic status. Likewise, boys with lowest LINE-1 methylation experienced an annual increase in the subscapular-to-triceps skinfold thickness ratio that was 0.07 Z/year (95% CI = −0.13, −0.01; *P = *0.03), or a raw ratio of approximately 0.02/year, greater than those in the upper three quartiles after adjustment for baseline age and socioeconomic status. Further adjustment for maternal BMI and plasma vitamin A did not change the direction, magnitude, or significance of the associations (data not shown). In addition, LINE-1 DNA methylation was inversely associated with change in waist circumference Z-score in a linear fashion (*P* trend = 0.02; **Table 2**). Boys in the lowest quartile of LINE-1 methylation experienced a 0.09 Z/year greater annual gain in waist circumference, or approximately 0.8 cm/year, than those in the highest quartile (*P* = 0.01).

**Table 2:** Change in adiposity indicators in 254 school-age boys from Bogotá, Colombia, according to quartiles of LINE-1 DNA methylation.

	Q1	Q2	Q3	Q4	*P* 1
	N = 64	N = 64	N = 63	N = 63	
**Median (Range) %5** **mC**	79.05 (76.26, 79.62)	80.05 (79.63, 80.58)	80.90 (80.59, 81.40)	82.20 (81.41, 85.24)	
**BMI-for-age Z-score** 2					
Baseline^3^	0.18±1.09	0.20±1.05	0.08±0.99	0.14±1.13	
Change (/year)^4^	0.04±0.02	−0.04±0.03	0.00±0.03	−0.02±0.02	0.24
Adjusted difference (95% CI)^5^	Reference	−0.08 (−0.16, 0.00)	−0.04 (−0.11, 0.03)	−0.06 (−0.12, 0.01)	0.21
**Waist Circumference Z-score** 6					
Change (/year)^4^	0.05±0.03	0.00±0.03	0.00±0.03	−0.03±0.02	0.03
Adjusted difference (95% CI)^5^	Reference	−0.05 (−0.14, 0.04)	−0.06 (−0.14, 0.02)	−0.09 (−0.17, −0.02)	0.02
**Skinfold Thickness Ratio Z-score** 6,7					
Baseline^3^	−0.03±0.91	0.13±0.77	0.28±0.84	0.03±0.95	
Change (/year)^4^	0.01±0.03	−0.07±0.03	−0.08±0.03	−0.03±0.02	0.32
Adjusted difference (95% CI)^5^	Reference	−0.08 (−0.16, 0.00)	−0.09 (−0.17, −0.01)	−0.04 (−0.12, 0.03)	0.28

1 For a test of linear trend when a variable that represented the median value of each quartile was introduced into a linear regression model as a continuous predictor (Wald test).

2 According to the World Health Organization growth reference for children 5–19 years [30].

3 Values are means ± SD.

4 Values are means ± SE.

5 Adjusted for baseline age and socioeconomic status.

6 Age-standardized using the LMS method with data for boys 5–16 years of age in NHANES III [32].

7 Subscapular-to-tricipital skinfold thickness ratio.

There were no significant associations between LINE-1 methylation and any of the adiposity indicators at baseline, or over the follow-up period among girls (**Table 3**). Global DNA methylation was not related to linear growth in boys or girls (**Table 4**).

**Table 3:** Change in adiposity indicators in 299 school-age girls from Bogotá, Colombia, according to quartiles of LINE-1 DNA methylation.

	Q1	Q2	Q3	Q4	*P* 1
	N = 75	N = 74	N = 75	N = 75	
**Median (Range) %5** **mC**	78.43 (75.16, 79.22)	79.65 (79.23, 79.96)	80.32 (79.97, 80.89)	81.52 (80.91, 85.69)	
					
**BMI-for-age Z-score** 2					
Baseline^3^	0.09±0.83	0.06±0.95	−0.04±0.91	0.09±0.99	
Change (/year)^4^	0.00±0.02	0.05±0.02	0.01±0.02	0.00±0.02	0.65
Adjusted difference (95% CI)^5^	Reference	0.05 (−0.01, 0.11)	0.00 (−0.06, 0.07)	−0.01 (−0.07, 0.06)	0.65
**Waist Circumference Z-score** 6					
Change (/year)^4^	0.00±0.02	0.00±0.03	0.03±0.03	−0.01±0.03	0.84
Adjusted difference (95% CI)^5^	Reference	0.01 (−0.06, 0.08)	0.04 (−0.03, 0.11)	−0.01 (−0.08, 0.06)	0.99
**Skinfold Thickness Ratio Z-score** 6,7					
Baseline^3^	0.03±0.72	−0.01±0.82	−0.09±0.61	0.02±0.57	
Change (/year)^4^	0.00±0.03	0.00±0.03	0.01±0.02	0.00±0.03	0.87
Adjusted difference (95% CI)^5^	Reference	0.00 (−0.08, 0.07)	0.01 (−0.05, 0.07)	0.00 (−0.07, 0.07)	0.85

1 For a test of linear trend when a variable that represented the median value of each quartile was introduced into a linear regression model as a continuous predictor (Wald test).

2 According to the World Health Organization growth reference for children 5–19 years [30].

3 Values are means ± SD.

4 Values are means ± SE.

5 Adjusted for baseline age and socioeconomic status.

6 Age-standardized using the LMS method with data for boys 5–16 years of age in NHANES III [32].

7 Subscapular-to-tricipital skinfold thickness ratio.

**Table 4:** Change in height-for-age in 553 school-age children from Bogotá, Colombia, according to quartiles of LINE-1 DNA methylation^1^.

	Q1	Q2	Q3	Q4	*P* 2
**Boys**					
*N*	64	64	63	63	
Median (Range) %5 mC	79.05 (76.26, 79.62)	80.05 (79.63, 80.58)	80.90 (80.59, 81.40)	82.20 (81.41, 85.24)	
Baseline height-for-age Z-score^3^	−0.84±1.01	−0.96±0.96	−0.81±0.90	−0.79±0.78	
Change (/year)^4^	0.04±0.02	0.05±0.02	0.04±0.02	0.06±0.02	0.48
Adjusted difference (95% CI)^5^	Reference	0.01 (−0.05, 0.08)	0.00 (−0.06, 0.06)	0.02 (−0.03, 0.08)	0.55
**Girls**					
*N*	75	74	75	75	
Median (Range) %5 mC	78.43 (75.16, 79.22)	79.65 (79.23, 79.96)	80.32 (79.97, 80.89)	81.52 (80.91, 85.69)	
Baseline height-for-age Z-score^3^	−0.73±0.90	−0.61±1.19	−0.73±0.88	−0.82±1.11	
Change (/year)^4^	0.06±0.02	0.03±0.02	0.03±0.02	0.08±0.02	0.54
Adjusted difference (95% CI)^5^	Reference	−0.02 (−0.08, 0.03)	−0.03 (−0.08, 0.03)	0.02 (−0.04, 0.08)	0.54

1 Height-for-age Z-scores were determined using the World Health Organization growth reference for children 5–19 years [30].

2 For a test of linear trend when a variable that represented quartiles was introduced into a linear regression model as a continuous predictor (Wald test).

3 Values are mean ± SD.

4 Values are mean ± SE.

5 Adjusted for baseline age and socioeconomic status.

## Discussion

In this study of 553 school children from low- and middle-income families in Bogotá, Colombia, we found inverse associations of LINE-1 methylation at time of recruitment into the cohort with annual change in three age- and sex-standardized measures of adiposity (BMI-for-age Z-score, waist circumference-for-age Z-score, and subscapular-to-triceps skinfold thickness ratio-for-age Z-score) among boys. Because mean BMI of this population at baseline was higher than the international growth reference [30], these associations likely reflect unhealthy gains in adiposity.

These findings contribute to the understanding of biological mechanisms that underlie childhood obesity. Early life environmental stimuli could induce long-lasting changes in DNA methylation profiles that are related to obesity and cardiometabolic disease. For example, middle-aged adults who were conceived during the Dutch Winter Famine exhibited persistent changes in methylation of genes involved in cardiometabolic diseases [33], [34] and had a higher BMI and waist circumference than their unexposed same-sex siblings [35]. The direct relation between DNA methylation and obesity has mostly been examined in cross-sectional studies of adults. A study conducted among middle-aged Samoan islanders reported that LINE-1 DNA methylation was positively correlated with BMI among women [8]. In a cohort of Singaporean Chinese adults, hypermethylation of the satellite (AS) repetitive element was associated with higher BMI at baseline in both men and women [11]. In the same study, higher AS methylation was related to incidence of cardiovascular disease over follow-up among men only [11]. The authors postulated that AS hypermethylation could serve as a biomarker of cardiovascular disease risk and obesity; yet, the relation between DNA methylation and BMI was assessed only at baseline, making it impossible to determine whether higher DNA methylation preceded high BMI or vice versa. Other cross-sectional studies have not found associations between DNA methylation and measures of adiposity in adults [10], [12], [13], [14], [36]. To date, the largest gap in this area of research is the lack of longitudinal investigations to assess the temporal relation of DNA methylation with changes in anthropometry. We were able to examine prospective changes in three indicators of adiposity with respect to LINE-1 DNA methylation quantified from blood samples collected at baseline from a large and representative sample of school-age children. Our results indicate that LINE-1 hypomethylation is related to adverse weight gain patterns during the school years. The fact that change in waist circumference was strongly associated with LINE-1 methylation is especially important because abdominal fat mass is an independent predictor of morbidity [37] and mortality [38] in adults. Furthermore, there is prospective evidence that accrual of central fat during childhood is related to adverse metabolic consequences in later life [39]. Our findings also provide a basis to further investigate molecular mechanisms involved in early life weight gain. Considering that adipocyte quantity is established sometime between late childhood and early adolescence [40], identifying modifiable pathways involved in adipogenesis during the school-age years would be particularly valuable to interventions efforts aimed at improving long term cardiometabolic health.

The trend between LINE-1 methylation and the adiposity indicators was apparent among boys only. It is uncertain whether the sex-specific association is due to a sex hormone effect on the relation between methylation and health outcomes, or to sex-specific weight gain patterns. We also noted that the association between LINE-1 DNA methylation and change in anthropometry was in the opposite direction of what was observed in adults [8], [11]. This discrepancy could be attributed to differences in study design, as the studies of adults were cross-sectional, which restricted inference on causation. There may also be inherent age-specific differences in epigenetic effects. For example, although studies in adults have reported inverse relations between age and LINE-1 methylation [10], [41], DNA methylation was not associated with age in our study population [25], or in a pilot study of girls 6–17 years in the US [42]. Because the epigenome reflects perinatal [43] and lifetime exposures [20], [44], [45], findings from children may not be directly comparable to adult studies. Nevertheless, longitudinal studies in both adults and children will be useful to disentangle the nature of the relation between repetitive element methylation and weight gain.

There are a few pathways that may explain the associations observed in boys. The variability in level of repetitive element methylation may reflect concomitant differences in gene-specific methylation, as there is evidence that some prenatal exposures are associated with both gene-specific and repetitive element DNA methylation [46]. Two recent prospective studies found significant associations between methylation of candidate genes quantified from cord blood and childhood body size [18], [47]. Godfrey et al. reported that hypermethylation of the retinoid X receptor alpha (*RXRA*) promoter region was correlated with greater adiposity at age 9 in two independent birth cohorts [47]. Because *RXRA* regulates transcriptional activity through heterodimerization with peroxisome proliferator-activated receptors (*PPARs*) directly involved in regulating insulin sensitivity and fat metabolism [48], hypermethylation silencing of *RXRA* could lead to weight gain. This hypothesis is consistent with the observation that *RXRA* expression in adipose tissue of obese mice and humans is diminished [49]. There is also increasing evidence from animal models that exposure to ubiquitous obesogenic chemicals, such as Bisphenol-A (BPA), can influence DNA methylation [3] and promote adipogenesis [50]. An *in vitro* study that examined omental adipose tissue biopsies from children demonstrated that exposure to environmentally relevant levels of BPA increased expression of *PPAR*-γ [51], a nuclear hormone receptor that stimulates fat cell differentiation. Taken together, these findings support the notion that DNA methylation and gene expression changes could precede alterations in body composition. Whether modification of DNA methylation profiles through dietary interventions influences body composition deserves further investigation.

We did not find any associations between LINE-1 methylation and linear growth. The lack of association could be related to the age distribution of the study population. Preliminary results in a U.S. birth cohort suggested that higher cord blood LINE-1 methylation was related to greater childhood height up to 7 years among boys [52]. However, the school-age years coincide with the pre-pubertal nadir, where linear growth velocity decelerates and reaches a minimum approximately two years prior to the adolescent height spurt, which occurs around 14 years in boys and 12 years in girls [53]. Given that mean age at baseline in our cohort was 8.8 years, the follow-up period may not have been sufficient to capture substantial changes in height. Long-term cohort studies examining the relation of DNA methylation with linear growth beyond adolescence are warranted.

Our study has several strengths. We were able to examine LINE-1 methylation in a large and representative sample of children from a setting where the increasing prevalence of childhood obesity is becoming a serious public health problem. The prospective design and use of repeated anthropometric measures enhanced our ability to explore the temporal relation between LINE-1 methylation and changes in body weight. We also adjusted the estimates of association for key potential confounders, including age at recruitment and socioeconomic status. LINE-1 methylation was determined using pyrosequencing technology, a highly reproducible and accurate method to quantify DNA methylation, with all assays run in duplicate to minimize variability and enhance accuracy. Additionally, we used DNA from peripheral WBC, which is of high intrinsic value in epidemiologic studies as it is easily obtained and reflects systemic interindividual variation in germ-layer cells [54]. This study also has some limitations. First, we had only one measurement of LINE-1 methylation at the time of enrollment as the primary exposure. Second, we did not account for the proportion of white blood cell subtypes from the buffy coat in the analyses. There is some evidence that DNA methylation is inversely related to the percentage of lymphocytes [10]; whether differential leukocyte counts are associated with development of adiposity is a possibility that warrants further investigation. Third, although there is some evidence that tissue-specific methylation profiles are reflected in circulating cell epigenomes [55], additional research is required to determine whether LINE-1 methylation quantified from peripheral blood reflects methylation patterns in relevant target tissues, such as adipose tissue. Fourth, there is potential random measurement error in anthropometry. Finally, generalizability to other ethnicities may be limited, as there is some evidence that Hispanics may have lower LINE-1 methylation than non-Hispanic whites [12].

We conclude that lower LINE-1 methylation is related to development of adiposity in school-age boys. Considering the role of diet in DNA methylation pathways [56] and in light of evidence that LINE-1 methylation is responsive to external cues in the short term [20], these findings point toward the need for longitudinal studies to investigate the effect of dietary interventions on repetitive element methylation patterns and subsequent anthropometric changes.
